# Association of HbA1c and cardiovascular and renal disease in an adult Mediterranean population

**DOI:** 10.1186/1471-2369-14-151

**Published:** 2013-07-17

**Authors:** Domingo Hernandez, Ana Espejo-Gil, M Rosa Bernal-Lopez, Jose Mancera-Romero, Antonio J Baca-Osorio, Francisco J Tinahones, Ana M Armas-Padron, Pedro Ruiz-Esteban, Armando Torres, Ricardo Gomez-Huelgas

**Affiliations:** 1 Nephrology Department, Hospital Regional Universitario Carlos Haya, Malaga, Spain; 2 Internal Medicine Department, Hospital Regional Universitario Carlos Haya, Avenida Carlos Haya s/n, 29010 Malaga, Spain; 3 Biomedical Research Laboratory, Endocrinology Department, Hospital Virgen de la Victoria, Campus de Teatinos s/n., 29010 Malaga, Spain; 4 Ciber Fisiopatologia de la Obesidad y Nutricion, Instituto de Salud Carlos III, Madrid, Spain; 5 Health Care “Ciudad Jardin”, Malaga, Spain; 6 Endocrinology Department, Hospital Virgen de la Victoria, Malaga, Spain; 7 Health Care “La Cuesta”, Tenerife, Spain; 8 Research Unit, Hospital Universitario de Canarias, Tenerife, Spain

**Keywords:** Glycated hemoglobin, Chronic kidney disease, Cardiovascular disease

## Abstract

**Background:**

Increasing evidence suggests a mechanistic link between the glycemic environment and renal and cardiovascular events, even below the threshold for diabetes. We aimed to assess the association between HbA1c and chronic kidney disease (CKD) and cardiovascular disease (CVD).

**Methods:**

A cross-sectional study involving a random representative sample of 2270 adults from southern Spain (Malaga) was undertaken. We measured HbA1c, serum creatinine and albuminuria in fasting blood and urine samples.

**Results:**

Individuals without diabetes in the upper HbA1c tertile had an unfavorable cardiovascular and renal profile and shared certain clinical characteristics with the patients with diabetes. Overall, a higher HbA1c concentration was strongly associated with CKD or CVD after adjustment for traditional risk factors. The patients with known diabetes had a 2-fold higher odds of CKD or CVD. However, when both parameters were introduced in the same model, the HbA1c concentration was only significantly associated with clinical endpoints (OR: 1.4, 95% CI, 1.1-1.6, *P* = 0.002). An increase in HbA1c of one percentage point was associated with a 30% to 40% increase in the rate of CKD or CVD. This relationship was apparent in persons with and without known diabetes. ROC curves illustrated that a HbA1c of 37 mmol/mol (5.5%) was the optimal value in terms of sensitivity and specificity for predicting endpoints in this population.

**Conclusion:**

HbA1c levels were associated with a higher prevalence of CKD and CVD cross-sectionally, regardless of diabetes status. These data support the value of HbA1c as a marker of cardiovascular and renal disease in the general population.

## Background

Chronic kidney disease (CKD), evidenced by either albuminuria or decreased kidney function, is increasing worldwide, especially in individuals with metabolic abnormalities, including dysglycemic disorders [1,2]. In addition, much research has demonstrated a significant relationship between components of the cardiometabolic syndrome and both CKD and cardiovascular disease (CVD), irrespective of the presence of diabetes mellitus or hypertension [3-6]. Given this background, a mechanistic link between metabolic abnormalities and adverse renal and cardiovascular events seems likely.

The glycated hemoglobin concentration (HbA1c) is a useful indicator of mean blood glucose concentrations over the preceding 3 months, but it may also unmask dysglycemic disorders, including the prediabetic state. Epidemiological studies have reported that higher HbA1c values were strongly associated with microvascular diabetic complications, and CVD and all-cause mortality in populations with and without diabetes [7-11]. However, the linear relationship between HbA1c concentrations and the presence of CKD in the general population has not been fully elucidated, even when a prediabetes condition could be present [12,13]. A high cardiovascular morbidity and mortality has been associated with albuminuria and/or reduced kidney function, regardless of age [14]. Thus, screening of HbA1c concentrations in the general population could be crucial to identify different HbA1c phenotypes associated with CKD and CVD, even in the absence of diabetes.

Cardiovascular morbidity and mortality rates are not uniform throughout European countries. A higher prevalence of cardiovascular risk factors, especially dyslipidemia and obesity, has been reported in southern Spain [15,16].

The aim of this study, therefore, was to assess the relationship between HbA1c concentrations and CKD and CVD in an adult urban Mediterranean population from southern Spain (Malaga).

## Methods

This cross-sectional, analytical, epidemiological study was derived from the IMAP study (Multidisciplinary Intervention in Primary Care) and involved a random representative sample of 2270 adults (18–80 years) assigned to a specific Health Center (n: 29,818 people) in Malaga city (Spain) in order to determine a HbA1c cut-off point for CKD and CVD. The methodology of the IMAP study has been reported previously [16,17]. The participants were located and invited by telephone to participate in the study. Recruitment was done between January and June 2007. All the participants underwent a clinical interview and physical examination, and provided fasting (≥12 hours) blood and urine samples. The blood and urine samples were analyzed in the reference hospital laboratory (Carlos Haya University Hospital) and biochemical determinations were assayed using conventional techniques. All the clinical information and laboratory values were contrasted with chart review over the previous three months. HbA1c was measured by high performance liquid chromatography (Adams A1C, HA-8160, ARKRAY Kyoto, Japan) and standardized according to the criteria of the National Glycohemoglobin Standardization Program [18].

Fasting plasma glucose levels of <5.6 mmol/L, 5.6-6.9 mmol/L, and ≥7.0 mmol/L were considered as normal, impaired fasting glucose, and diabetes, respectively. Participants were considered to be patients with diabetes if they had a previous diagnosis of diabetes, were taking drugs for diabetes, or their HbA1c was ≥48 mmol/mol (≥6.5%) [19]. CVD was considered to be the presence of a previous history of coronary heart disease, heart failure, cerebrovascular disease, aortic aneurysm, or peripheral arterial disease. The glomerular filtration rate (GFR) was estimated using the Chronic Kidney Disease Epidemiology Collaboration (CKD-EPI) equation [20]. Urine albumin and creatinine were measured from a spot urine sample. Albuminuria was defined as an albumin-to-creatinine ratio ≥33.8 mg/mmol. Individuals with an estimated GFR below 60 ml/min/1.73 m^2^ and/or albumin-to-creatinine ratio ≥33.8 mg/mmol were considered to have CKD [21].

### Other variables of interest

Blood samples were assayed for total and low-density lipoprotein cholesterol and uric acid. The body mass index (BMI) was obtained from measured height and weight. Obesity was defined as a BMI > 30 kg/m^2^. Information on cigarette smoking was also elicited during the interview. Resting systolic blood pressure was measured using a random-zero sphygmomanometer. As the study sample was not selected, hypertension was defined as a systolic blood pressure ≥140 mmHg and/or a diastolic blood pressure ≥90 mmHg, or treatment with antihypertensive drugs.

The study was approved by the Ethics and Research Committees of Carlos Haya Hospital, Virgen de la Victoria Hospital, Canary Isles University Hospital, Ciudad Jardin Health Centre, and La Cuesta Health Centre, and informed consent was obtained from all the participants. The study was conducted in accordance with the Declaration of Helsinki. In addition, reporting of the study conforms to the STROBE statement along with references to STROBE and the broader EQUATOR guidelines [22].

### Statistical analysis

HbA1c values were divided into tertiles in order to analyze the demographic and clinical characteristics of the population. Quantitative variables are expressed as the mean ± standard deviation, whereas the qualitative variables are expressed as percentages. An analysis of variance (ANOVA) was used to compare quantitative variables and the *χ*^2^ test for qualitative variables. All probability values were two-tailed and all confidence intervals (CI) were computed at the 95% level. Logistic regression analysis was used to assess the relationship between CKD or CVD and HbA1c and diabetes. Age and gender-adjusted odds ratios were calculated in the entire population. A similar approach was performed after adjusting for other confounding variables. The Hosmer-Lemeshow goodness of fit was the principal criterion for selection of the final models. Receiver operating characteristic (ROC) curves were plotted and the area under the curve (AUC) was estimated to assess the predictive performance of HbA1c in discriminating CKD or CVD. In addition, the best discrimination limit for HbA1c levels was explored by the maximum of the Younden’s index (sensitivity + specificity – 1). This corresponds to the value where sensitivity plus specificity is maximized. All computations were carried out using SPSS 15.0 for Windows (SPSS Inc., Chicago, IL, USA) and Epidat 3.1. A *P* value <0.05 was considered significant.

## Results

Overall, CKD and CVD were documented in 5.2% and 4%, respectively, of the individuals without diabetes. These percentages were higher in the patients with known diabetes (19.4% and 15.4%, respectively).

Table 1 displays clinical and demographic characteristics for different HbA1c tertiles and known diabetes. As expected, patients with known diabetes showed a higher mean HbA1c concentration 56 mmol/mol (7.3 ± 1.4%) than the rest of the study sample 37 mmol/mol (5.5 ± 0.4%). They were also older and had a worse metabolic, cardiovascular and renal profile, evidenced by a higher BMI, blood pressure and plasma lipids, as well as a lower CKD-EPI than patients without diabetes. Indeed, cardiovascular disease and CKD were more frequent in the patients with diabetes. In addition, individuals in the upper HbA1c tertile (HbA1c > 39 mmol/mol; 5.7%) shared a few characteristics with the patients with diabetes.

**Table 1:** Clinical characteristics of the study population by HbA1c tertiles and known diabetes

**HbA1c tertiles**				
	**22-34 mmol/mol**	**36-39 mmol/mol**	**40-46 mmol/mol**	
	**(4.2-5.3%)**	**(5.4-5.7%)**	**(5.8-6.4%)**	**Known diabetes**
	**N = 807**	**N = 762**	**N = 500**	**N = 201**
Age, *y.*	36 ± 12	42.4 ± 14.5	51 ± 15	61 ± 12
Male gender, *n* (*%*)	375 (46.5)	390 (51)	364 (52.6)	103 (51)
Body mass index, *kg/m*^*2*^	25.4 ± 4	26.6 ± 5	28.3 ± 5	30.4 ± 5
Systolic blood pressure, *mmHg*	121 ± 15	125 ± 16	128 ± 16	134 ± 18
Diastolic blood pressure, *mmHg*	72 ± 9	75 ± 10.4	77 ± 10	77 ± 10
Serum creatinine, *μmol/l*	68 ± 17.5	69.8 ± 17.5	70.7 ± 17.5	74.3 ± 25
CKD-EPI, *ml/min/1.73 m*^*2*^	109 ± 17	104 ± 18	98.5 ± 18	86 ± 19
FPG, *mmol/mol*	4.7 ± 0.5	4.9 ± 0.5	5.2 ± 0.6	8.1 ± 0.4
HbA1c, *mmol/mol (%*)	32 (5.1 ± 0.16)	36 (5.5 ± 0.11)	41 (5.9 ± 0.17)	56 (7.3 ± 1.4)
Total cholesterol, *mmol/l*	3.6 ± 2	5.2 ± 1.1	5.4 ± 1	5.5 ± 1.1
LDL-cholesterol, *mmol/l*	3 ± 0.8	3.3 ± 0.9	3.5 ± 0.9	3.5 ± 0.9
Triglycerides, *mmol/l*	1 ± 0.6	1.2 ± 0.8	1.3 ± 0.9	1.7 ± 1
Uric acid, *mmol/l*	261.7 ± 77.4	279.6 ± 83.3	291.5 ± 77.3	309.3 ± 97.5
Urinary albumin/creatinine, *mg/mmol*	10.5 ± 49.7	9.8 ± 36.2	11.3 ± 21.5	24.8 ± 63.3
Dyslipidemia, *n (%)*	51 (6.3)	96 (12.6)	98 (20)	72 (36)
Hypertension, *n (%)*	66 (8.2)	110 (14.4)	123 (24.6)	104 (52)
Smoking, *n (%)*	227 (28)	222 (29)	133 (26.6)	46 (23)
Cardiovascular disease, *n (%)*	13 (1.6)	17 (2.2)	33 (6.6)	31 (15.4)
CKD, *n (%)*	33 (4.1)	47 (6.2)	60 (12)	39 (19.4)

Values are expressed as mean ± SD. *P* < 0.001 for differences between HbA1c tertiles for all variables except for smoking and urinary albumin/creatinine.

HbA1c values are expressed in IFCC (International Federation of Clinical Chemistry) units (mmol/mol- no decimal point) followed by NGSP (National Glycohemoglobin Standardization Program) units (%-one decimal). Conversion from IFCC to NGSP units is provided by the equation: NGSP = [(0.09148 ∗ IFCC) + 2.152].

*Abbreviations*: CKD-EPI, Chronic Kidney Disease Epidemiology Collaboration equation; FPG, fasting plasma glucose; LDL, low density lipoprotein, CKD, chronic kidney disease.

Table 2 shows the relationship between HbA1c and CKD and/or CVD after adjustment for age and gender only, and then after adjustment for age, gender and other risk factors. Known diabetes was significantly associated with CKD, CVD, or both, with approximately 2-fold increases. These odds were only slightly attenuated after adjustment for known risk factors. HbA1c levels were also independently related to clinical endpoints after adjustment for traditional risk factors. However, when known diabetes and HbA1c levels were introduced in the same model, diabetes was not significantly associated with CKD or CVD. No interaction was found between HbA1c concentration and gender (*P* = 0.503). Of note, an increase in HbA1c levels of one percentage point was associated with a 30% to 40% increase in CKD or CVD. Thus, the significant associations between diabetes and CKD and CVD seemed to be entirely mediated by the HbA1c concentration, irrespective of the diabetic status. Similar significant associations were found between HbA1c levels and the clinical endpoints (CKD or CVD) in the diabetic (odds ratio 1.03, 95% CI 1.03-1.6, *P* = 0.025) and non-diabetic populations (odds ratio 1.1, 95% CI 1.1-3.4, *P* = 0.010). Finally, fasting glucose levels were similarly associated with CKD or CVD (odds ratio 1.011, 95% CI 1.005-1.017, *P* < 0.001) when they were entered into the multivariate model instead of the HbA1c concentration.

**Table 2:** Logistic regression analysis for chronic kidney disease and cardiovascular disease in the entire study population based on known diabetes and HbA1c (per 1-percentage point increase)

**Dependent variable**	**Independent variables**	**Age and sex-adjusted odds ratio (95% CI)**	***P *value**	Age, sex and other factor-adjusted* odds ratio (95% CI)	***P *value**
CKD					
	Model 1: HbA1c	1.4 (1.2-1.7)	0.000	1.4 (1.2-1.8)	0.000
	Model 2: Known diabetes	2.8 (1.8-4.4)	0.000	2.5 (1.5-4)	0.000
	Model 3: HbA1c &	1.2 (0.97-1.5)	0.110	1.3 (0.99-1.6)	0.057
	known diabetes	2.1 (1.2-3.7)	0.013	1.7 (0.96-3.3)	0.067
CVD					
	Model 1: HbA1c	1.4 (1.2-1.6)	0.000	1.4 (1.1-1.6)	0.000
	Model 2: Known diabetes	1.9 (1.2-3.2)	0.007	1.7 (1.02-2.8)	0.041
	Model 3: HbA1c &	1.3 (1.1-1.7)	0.012	1.4 (1.1-1.8)	0.009
	known diabetes	1.2 (0.6-2.3)	0.582	0.9 (0.5-1.9)	0.955
CKD or CVD					
	Model 1: HbA1c	1.5 (1.3-1.7)	0.000	1.5 (1.3-1.7)	0.000
	Model 2: Known diabetes	2.6 (1.8-3.8)	0.000	2.3 (1.5-3.3)	0.000
	Model 3: HbA1c &	1.3 (1.1-2.6)	0.004	1.4 (1.1-1.6)	0.002
	known diabetes	1.6 (0.9-2.7)	0.053	1.3 (0.8-2.3)	0.250

*Other factors include blood pressure, body mass index, smoking, dyslipidemia, uric acid, and cardiovascular disease.

*Abbreviations*: CKD, chronic kidney disease; CVD, cardiovascular disease.

**Figure 1:**
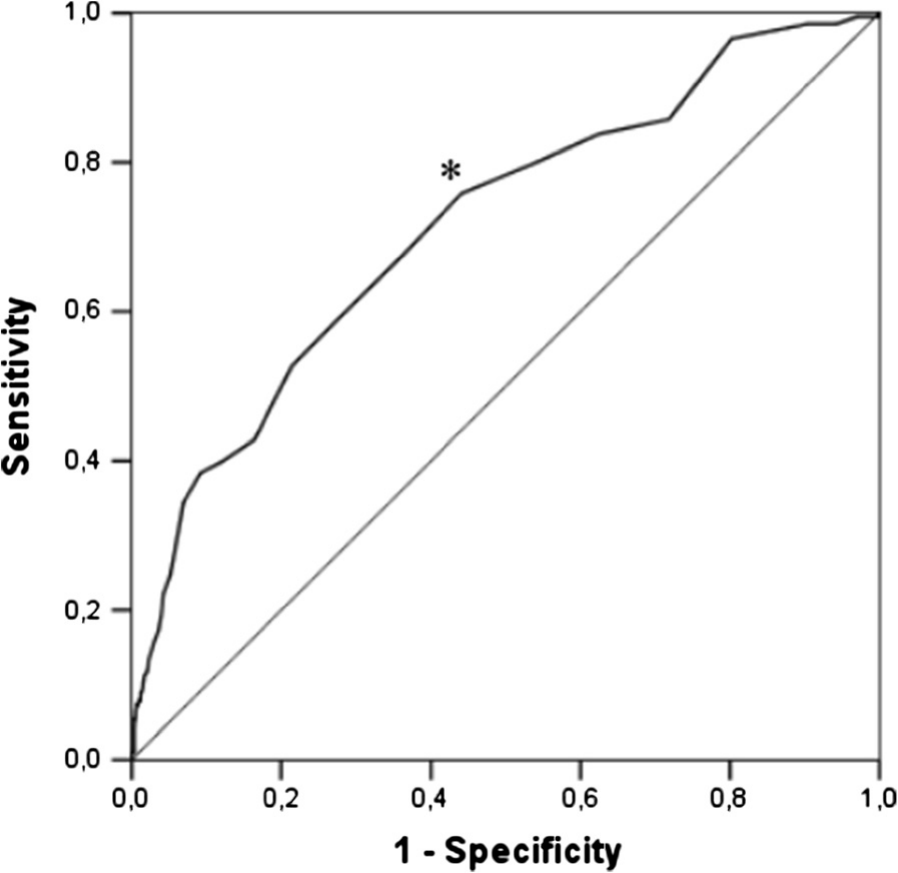
**ROC curve for HbA1c as a predictor of chronic kidney disease or cardiovascular disease in the entire study population. **The optimal predictor cut-off value (*) was that of the highest sensitivity together with the lowest number of false positives (specificity). This value corresponds to 37 mmol/mol (5.5%). ROC curve area: 0.76 (95% CI, 0.71-0.80).

Figure 1 shows the ROC curve for HbA1c levels and the endpoints in the entire study population. The AUC for the HbA1c concentration to predict CKD or CVD was 0.76 (95% CI, 0.71-0.80). The best Younden’s index was 0.37 for the HbA1 cut-off levels. The optimal value for HbA1c was 37 mmol/mol (5.5%), which gave a sensitivity of 82% and a specificity of 55%. We performed an additional analysis in individuals without diabetes (n = 2069). An HbA1c of 37 mmol/mol (5.5%) had less sensitivity (73%) and a similar specificity (60%) for clinical endpoints.

## Discussion

This study provides evidence for concluding that HbA1c may be a useful biomarker to identify individuals at risk for cardiovascular and renal disorders in the general population. Indeed, in this adult Mediterranean community-based population, we elucidated an HbA1c phenotype associated with CKD and CVD, even below the threshold commonly accepted for the diagnosis of diabetes. Similar associations were observed with fasting glucose levels, but HbA1c strongly correlates with the level of ambient glycemia during a 2 to 3 month period, so that it reflects the usual daily fasting and postprandial glucose levels as established by the American Diabetes Association (ADA) [19]. Since a high prevalence of cardiovascular risk factors has been recently reported in this particular population [15,16], these findings may be crucial for implementing prophylactic interventions in order to prevent or delay CKD and CVD.

Previous studies have reported that higher values of HbA1c are a powerful predictor of CVD and all-cause mortality in populations with and without diabetes [7-10]. In addition, a poor glycemic control, evidenced by higher HbA1c levels, has been associated with CKD in patients with diabetes, even in the absence of albuminuria and retinopathy [10,11]. However, whether the HbA1c concentration is related to the presence of CKD in the general population, regardless of the diabetic status, has been explored less [12,13].

In our study, we observed a strong and linear cross-sectional association between the HbA1 concentration and CKD and CVD. An increase in HbA1c levels of one percentage point was associated with a 30% to 40% increase in CKD or CVD*.* This association was independent of traditional factors associated with CKD and CVD, including diabetic status. Moreover, higher HbA1c levels were more strongly associated with the clinical endpoints other than diabetes when a composite endpoint (CKD or CVD) was considered in our multivariate analysis. Similar statistical associations were also observed in the subsets of diabetic and non-diabetic patients. It is plausible, therefore, that the increase in these clinical endpoints seemed to be mediated by the HbA1c level. In other words, the degree of hyperglycemia, rather than the presence or absence of diabetes as such, could be related to future cardiovascular or renal events. As an example, a HbA1c level ≤48 mmol/mol (6.5%) in an individual without diabetes may predict a higher likelihood of CKD or CVD than a HbA1c level of 37 mmol/mol (5.5%) in a patient with well-controlled diabetes. Further prospective studies are needed to clarify this issue.

Given our results, a lower threshold HbA1c concentration for detecting CKD or CVD seems likely. A HbA1c cut-off point of 37 mmol/mol (5.5%) proved to be the optimal predictor of composite endpoints in our study population, similar to that reported in community-based populations of adults without diabetes [8,9]. This cut-off value is close to the 39 mmol/mol (5.7%) figure, which has been retained by the ADA for defining the persons at high risk for developing type 2 diabetes [19]. In addition, our individuals without diabetes in the higher HbA1c tertile showed a worse cardiovascular and renal profile than the rest and shared various clinical characteristics of the metabolic syndrome with the patients with diabetes [17]. Given that the upper HbA1c limit in these patients was 6.4%, we cannot rule out that a prediabetic status could have a negative impact in our study, as reported by others [8]. Our findings suggest that concomitant metabolic risk factors may partly explain some of the mechanisms associated with CKD or CVD in this population from southern Spain. The fact that an increasing prevalence of metabolic risk factors has been strongly associated with a higher prevalence of CKD and CVD in the general population supports this hypothesis [3,4,23]. Whatever the case, whether lowering HbA1c decreases cardiovascular and renal events in this high-risk population deserves to be investigated by clinical trials.

A mechanistic relation between cardiorenal events could be established in these patients. The presence of insulin resistance and multiple risk factors has been associated with CKD in the general population without diabetes [24]. A growing body of evidence suggests that several pathophysiologic pathways may mediate the toxic effects of glucose fluctuations. The glycemic environment has been found to be proatherogenic through chronic inflammation [25,26]. Furthermore, HbA1c may be a link between chronic hyperglycemia and oxidative stress and endothelial dysfunction. HbA1c is a target for intracellular glycoxidation and peroxidation reactions that result in the formation of advanced glycation end products [27], which are implicated in the initiation and progression of atherosclerosis. Lastly, changes in glycemia may induce activation of growth factors, which may contribute to the development of intraglomerular hypertension and renal injury [28]. Taken together, these metabolic pathways may explain the relationship between HbA1c and microvascular and macrovascular complications in the general population, including persons with a prediabetic status. Whether new markers of glycemia, such as glycated albumin, may reflect a stronger association remains to be determined.

This study has a few limitations. The cross-sectional design of the study does not allow us to elucidate the temporal direction of the associations observed between HbA1c and CKD and CVD, nor the exposure risk, which would be better evaluated with a longitudinal study. Thus, whether HbA1c concentrations and CKD and CVD are causally related cannot be concluded from this cross-sectional study. In addition, the prevalence rates of CKD and CVD seen in this study were low. However, our study involved a random and representative sample of the adult population from southern Spain. Future longitudinal studies derived from this study should clarify this point. Secondly, the population studied was exclusively Caucasian; thus, the results cannot be extrapolated to other populations with differing diets or lifestyles. Finally, we only performed a single blood extraction for the diagnosis of diabetes based on glycemic indexes. Nevertheless, as the short-term reproducibility of HbA1c levels is very high [29], a second measurement would not provide a significantly different result. In addition, the interpretation of an isolated measurement of fasting plasma glucose and HbA1c values represents daily practice in clinical decision making and has been used in other population studies [7-9].

## Conclusion

In conclusion, this community-based epidemiological study highlights the independent association between HbA1c levels and CKD and CVD, regardless of the diabetes status. Very small shifts in the average HbA1c level of the general population could significantly affect the future incidence of cardiovascular and renal events in longitudinal studies. These data add to the evidence supporting screening of the HbA1c concentration in the general population to identify cut-off points associated with CKD and CVD in the clinical setting, even in the absence of diabetes.

## Competing interests

The authors declare that they have no competing interests.

## Authors’ contributions

DH. Wrote manuscript, contributed to discussion and reviewed/edited manuscript. AEG. Researched data and wrote manuscript. MRBL. Researched data and reviewed/edited manuscript JMR. Researched data and contributed to discussion. AJBO. Researched data and contributed to discussion. FJT. Contributed to discussion and reviewed/edited manuscript. AMAP. Wrote manuscript, and contributed to discussion. PRE. Contributed to discussion. AT. Wrote manuscript, and contributed to discussion. RGH. Wrote manuscript, contributed to discussion and reviewed/edited manuscript. All authors read and approved the final maunscript.

## Pre-publication history

The pre-publication history for this paper can be accessed here:

http://www.biomedcentral.com/1471-2369/14/151/prepub
